# Mitigating Financial Barriers to Trauma Care in Cameroon Through Locally Designed Quality Improvement

**DOI:** 10.1002/wjs.70495

**Published:** 2026-07-14

**Authors:** Dennis J. Zheng, Mirene Tchekep, Jean Baptiste Boumsong, Jean Gustave Tsiagadigui, Odette Dzemo Kibu, Fanny Nadia Dissak‐Delon, Rasheedat Oke, Catherine Juillard, Alain Chichom‐Mefire, S. Ariane Christie

**Affiliations:** ^1^ Program for the Advancement of Surgical Equity (PASE) Department of Surgery University of California Los Angeles California USA; ^2^ Edea Regional Referral Hospital Edea Cameroon; ^3^ University of Buea Buea Cameroon

**Keywords:** emergency care, health disparity, health economics, sub‐Saharan Africa, traumatic injury

## Abstract

**Background:**

In low‐ and middle‐income countries, trauma patients frequently face delays and inequities in care due to out‐of‐pocket payment requirements for basic medical supplies. In Cameroon, such financial barriers may impede timely emergency treatment and worsen outcomes. We evaluated whether the introduction of a locally designed care kit that provided essential supplies to emergency department (ED) providers without upfront payment improved timeliness and perceived affordability of initial trauma care.

**Methods:**

We conducted a pre‐/post‐observational cohort study using data from the Cameroon Trauma Registry at a public regional referral hospital. Injured patients presenting during the six months before emergency kit implementation (December 2021 through May 2022) were compared with those presenting during the six months after implementation (June through November 2022). Outcomes included ED treatments, time from presentation to disposition, ED disposition, and patient‐reported financial interference with care. Secondary analyses examined inpatient surgical care and discharge outcomes.

**Results:**

A total of 399 patients were included, of whom 208 (52%) presented after kit implementation. Demographics and injury characteristics were similar between groups. Post‐implementation, patients experienced shorter median time to ED disposition (13.8 vs. 15.1 h, *p* = 0.001) and were less likely to leave against medical advice (26.4% vs. 39.3%, *p* = 0.006). Although median estimated ED treatment costs increased (41,500 vs. 30,000 CFA francs, *p* < 0.001), the proportion of patients reporting financial interference with care decreased by more than half (22.6% vs. 47.6%, *p* < 0.001). Kit implementation did not reduce financial barriers to inpatient surgical care.

**Conclusions:**

Provision of essential emergency supplies without upfront payment was feasible and associated with improved timeliness and perceived affordability of trauma care. Addressing acute financial barriers in emergency settings may represent a pragmatic strategy to enhance access to care in resource‐limited environments.

## Graphical abstract

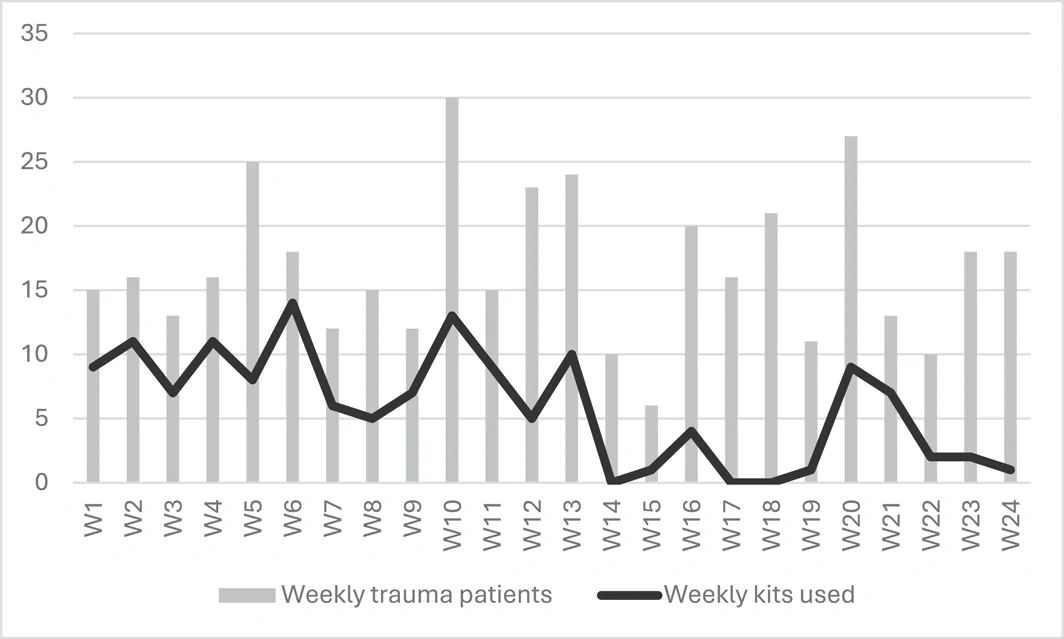

## 1 Introduction

The global burden of trauma falls disproportionately on low‐ and middle‐income countries (LMICs), where patients are the most vulnerable to financial hardship [1]. Especially in these settings, improving access to timely and equitable emergency care is a critical strategy for reducing injury‐related morbidity and mortality [2].

Because many LMICs have yet to fully implement universal health coverage, patients presenting with injuries face highly variable care access and costs [3]. In Cameroon, the overwhelming majority of trauma care is financed by out‐of‐pocket spending [4]. Patients presenting with injuries are commonly required to pay upfront for basic supplies used in their care, such as examination gloves or intravenous fluids. New patients or their family members are tasked with purchasing items at the hospital pharmacy or a nearby shop.

As noted in low‐resource settings around the world, policies that require injured patients to obtain and pay for materials used in their own care introduce financial disparities and treatment delays that can worsen outcomes [5]. Injured individuals could be forced to wait for their loved ones to gather funds before receiving life‐saving care or even depend on the generosity of hospital staff to finance necessary supplies. This risk of catastrophic health expenditure is already known to discourage Cameroonians from seeking care for their injuries [6].

Time‐related and monetary barriers to emergency care in Cameroon therefore represent crucial opportunities to improve the quality of available trauma care. At a referral hospital in the Littoral region, an international‐local collaboration developed a comprehensive trauma quality improvement (QI) bundle focused on standardizing care and strengthening clinical processes [7]. As part of this initiative, clinicians introduced an emergency kit system that made essential supplies immediately available in the emergency department (ED) without requiring upfront payment. In this study we aimed to assess whether implementation of the emergency kit was associated with improved timeliness and perceived affordability of initial trauma care.

## 2 Methods

### 2.1 Study Design

We conducted a pre‐post observational cohort study using data in the Cameroon Trauma Registry (CTR), a prospectively collected hospital‐based trauma registry.

### 2.2 Setting

The intervention was implemented at a public regional referral hospital in the Littoral region of Cameroon, one of four data collection sites in the CTR. Serving a catchment area of approximately 300,000 people, the hospital is located along Cameroon's busiest highway and frequently treats victims of road traffic injury (RTI). At the time of this study, it contained about 100 inpatient beds, two operating rooms, an intensive care unit, and an emergency department (ED) with over 3000 patient encounters in 2021. The ED was staffed by a group of rotating medical doctors and full‐time nurses, with consultation services available from general surgeons, orthopedic surgeons, and anesthesiologists.

### 2.3 Participants and Data Sources

The CTR is a prospectively collected hospital‐based trauma registry overseen by the University of Buea in collaboration with the University of California, Los Angeles, with data collected at four hospitals in Cameroon since 2011 [8]. Included are patients presenting for care of an injury experienced within the last 2 weeks who are either (1) formally admitted, (2) transferred or leave against medical advice (AMA), or (3) deceased upon arrival. Patients are followed from ED presentation through hospital exit. Trained research assistants obtain informed consent, interview staff and patients, and cross‐reference clinical records to ensure data accuracy. Core variables include demographics, injury mechanism, severity as measured by Abbreviated Injury Scale, treatments, disposition, estimated cost of care, and whether cost interfered with treatment. The latter question is a binary (‘yes/no’) question assessed at the time of patient discharge or exit from the hospital and is reported by patients or their family members during the exit interview conducted by research staff. Data quality is ensured through serial audit.

### 2.4 Study Intervention

Beginning in 2019, the hospitals involved in CTR data collection participated in a QI committee reviewing adverse events and establishing preventability [9]. Afterward, the regional referral hospital in this study continued to progress QI efforts. Local administrative and clinical staff participated in focus group discussions over 1 month, using CTR data and clinical experience to identify care gaps and develop a multi‐pronged QI bundle [7]. Components of the bundle included a context‐specific trauma protocol, bedside trauma checklist, and staff training on protocol implementation.

A key problem noted by the group was the delay caused by injured patients having to obtain materials used in their own care, especially when they could not immediately afford to do so. In response, it was suggested that the hospital begin to store supplies needed for basic care in not only the pharmacy but also separately in the ED. Staff discussion generated a consensus list of materials necessary for initial care of the patient including intravenous fluid resuscitation and local wound care (Table 1). These supplies were placed into individual sealed bags, and each emergency kit was stored securely in a locked cabinet in the ED. Hospital administration agreed that the kits could be used for a patient's care without requiring pre‐payment; the kit's cost (approximately 5200 Central African Francs, or about 8.70 United States Dollars in 2022) was later added to a patient's hospital bill. Pharmacy staff replenished the supply of emergency kits on a weekly basis.

**TABLE 1:** Emergency kit components and estimated costs.

	Quantity	Unit cost (CFA)	Total cost for one kit (CFA)
Examination gloves (one pair)	5	200	400
Intravenous cannula (18 gauge)	2	250	40
Syringe (10cc)	2	75	16
Normal saline (500cc)	2	750	1500
Intravenous administration kit	2	250	500
Gauze compresses	1	700	700
Bandages	1	350	350
Crepe bandage	1	500	500
Total cost for one kit (CFA)			5200

### 2.5 Statistical Methods and Data Analysis

The emergency kits were introduced for usage beginning in June 2022 for a pilot period of six months. In this study we compare patterns of injury care and financial aspects of treatment for patients in the six months immediately before kit implementation (December 2021 through May 2022) with those of patients enrolled in the six months following kit implementation (June 2022 through November 2022). Primary outcomes of evaluation included patient demographics, injury information, and types of emergency treatment recommended and administered. The CTR was also used for analysis of time‐related variables (e.g., time from ED presentation to disposition) and patient/family perceptions regarding financial aspects of their care. Hospital staff kept a separate log of number of emergency kits used each week, as kits were occasionally used for patients presenting for injury care who did not necessitate hospital admission (and thus were not included in the CTR). This information is presented separately.

Continuous variables were assessed for normality using the Shapiro‐Wilk test. Variables that were not normally distributed are presented as median and interquartile range (IQR); normally distributed variables are presented as mean ± standard deviation (SD). Categorical variables are summarized as counts and percentages. Between‐group comparisons for continuous variables used the Wilcoxon rank‐sum test for non‐normal data and Student's t‐test for normally distributed data. Categorical variables were compared using the chi‐square test, with Fisher's exact test applied when any expected cell count was fewer than five. All statistical tests were two‐sided, and an overall alpha of 0.05 was considered significant. To account for multiple comparisons across primary outcomes, *p*‐values were adjusted using the Bonferroni method (adjusted *α* = 0.05/number of tests). Statistical analysis was performed using Stata version 17.

### 2.6 Ethics

Approval to conduct the study was granted by institutional review boards from the University of California, Los Angeles, and the University of Buea in Cameroon (IRB #: 19‐000086 and 2021/1506‐07/UB/SG/IRB/FHS), along with the Cameroon National Ethics Committee (N°2018/09/1094/CE/CNERSH/SP and No. 2022/03/1444/CE/CNERSH/SP). Patients or patient surrogates (e.g., parents or guardians for patients under age 21) enrolled in the CTR were approached by trained Cameroonian research assistants for informed verbal consent using an IRB‐approved script. Research assistants documented obtaining verbal consent on CTR forms. The need for written informed consent for adults and parents/guardians had been waived by the IRB.

## 3 Results

Of the total 399 patients who enrolled in the CTR at the site during the study period, 208 (52%) presented after implementation of the emergency kit. Baseline demographic and injury characteristics were comparable between the pre‐kit and post‐kit cohorts, with no significant differences observed (Table 2). Trauma patients were young (median age 32.0 years pre‐kit vs. 35.5 years post‐kit) and predominantly male (81.7% vs. 76.9%). Less than one‐quarter of each group (22.3% vs. 22.1%) had been transferred from another facility before receiving care. The most common injury mechanism was road traffic injury, affecting over two‐thirds of both cohorts (70.2% vs. 68.0%). Most patients (50.5% vs. 45.2%) were estimated to have at least one serious injury, classified as presence of an Abbreviated Injury Score above 2 for an anatomic region. Of the initial treatments available in the ED, administration of IV fluids was completed for most patients. The post‐kit group featured significantly increased rates of recommendation and performance of wound debridement or laceration repairs, but there were no significant changes in rates of recommendation or performance of blood transfusion. Patterns of ED disposition differed significantly across the cohorts, as patients in the post‐implementation group compared to those in the pre‐implementation group were significantly more likely to be discharged home (53.9% vs. 44.0%, *p* = 0.05) and less likely to leave AMA (26.4% vs. 39.3%, *p* = 0.01). The secondary outcome of time to disposition following presentation in ED was significantly less in the emergency kit group compared to the non‐kit group (median 13.8 h [4.3, 20.2] vs. 15.1 h [5.5, 31.6], *p* = 0.001).

**TABLE 2:** Patient demographic and injury characteristics.

	Pre‐kit *N* = 191	Post‐kit *N* = 208	*p*‐value
Age, median [IQR], years	32.0 (25.0, 44.0)	35.5 (25.0, 48.0)	0.13
Sex, male–*n* (%)	156 (81.7)	160 (76.9)	0.24
Transferred from outside facility–*n* (%)	42 (22.3)	46 (22.1)	0.57
Mechanism of injury			
Road traffic injury	134 (70.2)	140 (68.0)	0.54
Fall	22 (11.5)	23 (11.2)	0.88
Stab/cut	11 (5.8)	16 (7.8)	0.44
Blunt force	11 (5.8)	20 (9.7)	0.15
Other	13 (6.8)	7 (3.3)	0.12
Number of serious injuries			
Zero	40 (21.0)	53 (25.5)	0.28
One	96 (50.5)	94 (45.2)	0.31
Two or more	54 (28.4)	61 (29.3)	0.91
Treatment in ED			
IV Fluids recommended	178 (93.2)	183 (88.0)	0.26
IV Fluids received	178 (93.2)	184 (88.5)	0.10
Wound debridement or laceration repair recommended	91(47.6)	144 (69.2)	< 0.001
Wound debridement or laceration repair received	90 (47.1)	143 (68.8)	< 0.001
Blood transfusion recommended	13 (6.8)	15 (7.2)	0.82
Blood transfusion received	13 (6.8)	14 (6.7)	0.98
Disposition in ED			
Admitted to hospital	84 (44.0)	112 (53.9)	0.049
Left against medical advice	75 (39.3)	55 (26.4)	0.006
Transferred	27 (14.1)	36 (17.3)	0.39
Died	5 (2.6)	5 (2.4)	0.89
Time from presentation to disposition, median [IQR], hours	15.1 (5.5, 31.6)	13.8 (4.3, 20.2)	0.001

Financial aspects of emergency care differed across the cohorts (Table 3). Compared to the pre‐kit group, patients in the post‐kit group had a higher median estimated cost of ED treatment (41,500 CFA [30,000, 70,000] vs. 30,000 CFA [24,000, 50,000], *p* < 0.001) but were significantly less likely to report interference in their care by financial cost (22.6% vs. 47.6%*, p* < 0.001). Most patients in both groups financed care through self‐pay or family assistance (86.9% vs. 69.7%), with utilization of insurance relatively uncommon (8.4% vs. 13.5%).

**TABLE 3:** Financial aspects of emergency department care.

	Pre‐kit *N* = 191	Post‐kit *N* = 208	*p*‐value
Estimated cost of initial treatment, median [IQR], CFA	30,000 (24,000, 50,000)	41,500 (30,000, 70,000)	< 0.001
Care financed through self or family alone	166 (86.9)	145 (69.7)	< 0.001
Care financed through insurance	16 (8.4)	28 (13.5)	0.11
Cost of treatment interfered with care–*n* (%)	91 (47.6)	47 (22.6)	< 0.001

Of the initial 399 patients who presented to the ED during the study period, 196 patients underwent hospital admission and received inpatient care (Table 4). Inpatients in the post‐implementation group were less likely to have abdominal or orthopedic surgery recommended (32.1% vs. 57.1%, *p* < 0.001) and less likely to undergo an abdominal or orthopedic operation (15.2% vs. 42.9%, *p* < 0.001). The most common reason for surgery to not be performed was cost, affecting over half of the patients in each group (66.7% vs. 62.5%). Inpatient disposition tended to be similar across the two groups, with roughly two‐thirds of each group discharged home (77.4% vs. 65.2%). Compared to the pre‐kit group, patients in the post‐kit group were more likely to be discharged prematurely due to financial reasons (21.4% vs. 9.5%, *p* = 0.02), though there was no significant difference in the estimated cost of remaining care for these patients.

**TABLE 4:** Financial aspects of inpatient hospital care.

	Pre‐kit *N* = 84	Post‐kit *N* = 112	*p*‐value
Abdominal or orthopedic surgery recommended	48 (57.1)	36 (32.1)	< 0.001
Abdominal or orthopedic surgery performed	36 (42.9)	17 (15.2)	< 0.001
If abdominal or orthopedic surgery not performed, reason: Cost–*n* (%)	8 (66.7)	15 (62.5)	0.81
Inpatient disposition			
Discharged home	65 (77.4)	73 (65.2)	0.06
Left AMA	10 (11.9)	18 (16.1)	0.41
Transferred	6 (7.1)	12 (10.7)	0.39
Died	2 (2.4)	4 (3.6)	0.63
Patient discharged prematurely due to financial reasons	8 (9.5)	24 (21.4)	0.02
If premature discharge, estimated cost of remaining care, median [IQR], CFA	275,000 (200,000–600,000)	600,000 (500,000–600,000)	0.13

**FIGURE 1:**
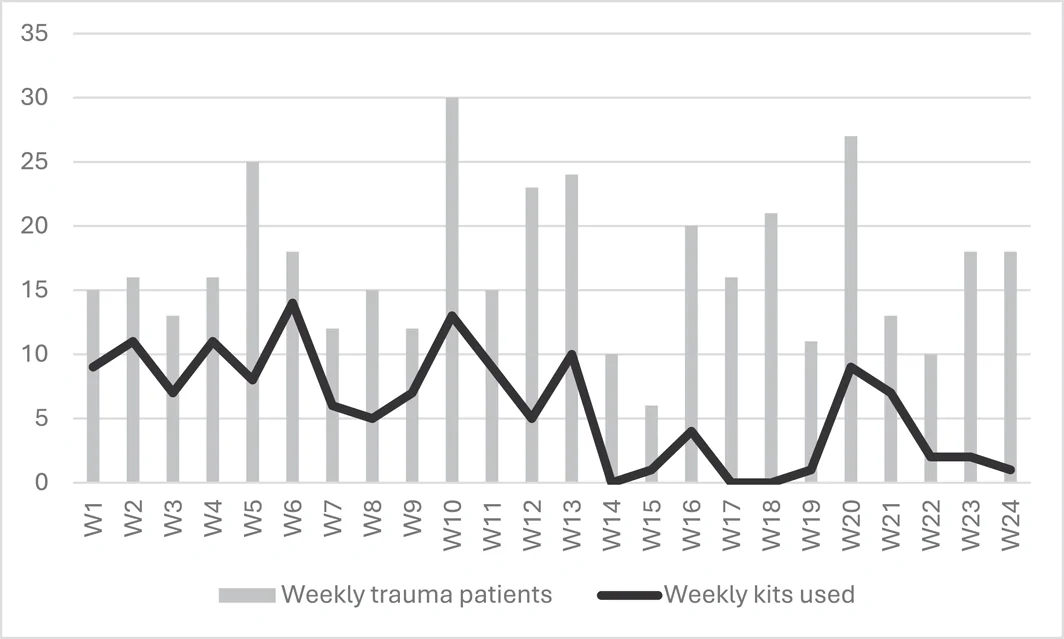
Trends in emergency kit usage by study week.

Kit usage fluctuated over time, ranging from a minimum of zero to maximum of 14 kits opened each week (Figure 1). Over the first half of the pilot period, the median number of kits used per week was 8.5 kits, tending to increase during weeks when more patients presented with injury. Most of these trauma patients were not admitted to the hospital and therefore were not eligible for inclusion in the CTR. Kit usage decreased over the second half of the pilot period as national supply chain issues and stock‐outs negatively affected the availability of kit components.

## 4 Discussion

In this single‐center pilot study, implementation of an emergency care kit providing essential supplies without upfront payment was associated with improved access to trauma care in a public hospital in Cameroon. Following kit introduction, patients experienced shorter time to ED disposition, lower rates of leaving AMA, and substantially reduced reports of financial interference with care.

Standardized tools for measuring emergency care capacity, such as the Hospital Emergency Unit Assessment Tool developed by the World Health Organization (WHO), evaluate the availability of key items ranging from airway devices to cervical collars [10, 11]. Many lists, however, under‐emphasize routinely used consumables—for example, examination gloves and intravenous fluids—which are frequently subject to stockouts or require out‐of‐pocket purchase in public facilities across Africa and Asia [12, 13]. In our experience, public hospital pharmacies in Cameroon store not only medications but also consumable items for patient care, where they can be securely held and easily sold to patients. This presents a potential target for trauma QI in these resource‐limited settings. When items commonly used in trauma care are stocked within the ED, they become physically more accessible to providers and less challenging for patients to obtain, rendering injury care more efficient.

High‐quality emergency care is contingent upon reliable procurement and supply chain management [14]. Shortages of physical resources and consumables represent critical barriers to care around the African continent, where trauma systems are largely underdeveloped and underfunded [15, 16, 17]. Universal health coverage has been proposed as a means of reducing the financial toxicity associated with trauma, but there is a dearth of data about costs incurred by patients when seeking injury care [18]. A scoping review of emergency care access measures found that affordability of care was severely understudied relative to other access dimensions such as distance and travel time [19]. Even when cost data is available, its variability is high [20]. In a WHO analysis, out‐of‐pocket health spending in Cameroon represented over 70% of overall health spending, the second‐highest rate among 47 African countries [21]. In contrast, a micro‐costing study of emergency care in Uganda observed that patients were rarely asked to spend out of pocket for basic supplies [22].

Finances heavily influence the provision of injury care in Cameroon, where per capita spending on health is just 240 United States Dollars, or just over 10% the global average [23]. Prior analysis of CTR data found that over 95% of patients paid out of pocket for injury care, less than 2% had access to insurance, and over half reported that cost served as a barrier to care [24]. In this study, rates of out‐of‐pocket spending were not as skewed, yet use of private insurance remained uncommon. Community‐based research has further emphasized the prominent role that perceptions of cost play in personal decision‐making after injury. A study of care‐seeking behavior in Southwest Cameroon noted that almost half of patients sustaining non‐fatal injuries chose not to seek formal care, often due to the requirement that they provide payment before receiving treatment [4]. In this context, it is highly encouraging that kit implementation was associated with over 50% reduction in patients reporting that cost interfered with their emergency care. Regardless, despite the significant effects of the emergency kit, over one quarter of ED patients left AMA in the intervention period. Discharge AMA, rates of which range from 0.07% to 20% in high‐income country EDs, has been linked to worsened health outcomes and increased health care costs [6]. In Cameroon this is likely exacerbated by the fact that patients unable to pay for care have been known to be detained by hospital authorities [25].

In recognition of the substantial role played by patient ability to pay for health care in Cameroon, government‐mandated programs for emergency payment deferral (EPD) have been established in several of the country's largest public hospitals. EPD allows patients who present in emergent situations to forgo payment for medications or services for the first 24 h of care, after which a formal invoice is created. Several studies have examined the effect of these programs, noting they allow for improved performance of diagnostic studies but do not necessarily improve access to surgery and are associated with increased costs of care [26, 27]. In similar fashion, the intervention described in this study shifted the timing of payment for medical supplies rather than lessened the actual costs of care. The kit's impact was limited to the ED, while access to inpatient treatments such as surgery remained dependent on patient finances. Consistent with prior EPD research, our study found that implementation of the emergency kit was associated with a significant increase in the reported cost of care. This change is likely attributable to multiple factors: an increase in patients choosing to remain at the hospital for care of their injuries, the cost of the emergency kits added to hospital bills, and the implementation of trauma protocols potentially encouraging more thorough initial workups. The reduction in reported financial interference, despite higher costs, likely reflects the changed timing and experience of payment. When supplies are immediately available, patients receive timely care without the stress of scrambling to gather funds. The financial obligation still exists but is deferred, reducing the acute perception that cost is interfering with care.

This study has several limitations. As a pilot, the intervention was not studied in a randomized controlled trial, making it difficult to ascertain its true effects. It was also introduced to the hospital simultaneously with other components of the trauma QI bundle, which included standardized trauma protocols and provider training, raising the possibility of confounding. Those other interventions, however, were focused on clinicians and unlikely to directly affect financial aspects of care. Finally, financial information such as the cost of care was derived from patient and provider estimates rather than official billing data, introducing potential measurement error.

Consistent with existing literature on LMIC QI projects, implementation of the emergency kit was facilitated by stakeholder engagement [28]. Hospital administration supported the idea, which had been generated through consensus discussion among staff. Even with substantial buy‐in, kit usage decayed over the 6‐month pilot period, as the availability of its components was not reliable. Further research into the processes of supply procurement is necessary to maximize the sustainability of such improvements in care.

## 5 Conclusion

Our study demonstrates that relatively simple measures to offset acute care costs for injured patients in the ED are feasible to implement and have the potential to increase access to care in Cameroon and similar resource‐limited settings where finances represent a critical barrier.

## Author Contributions

**Dennis J. Zheng:** conceptualization, methodology, formal analysis, investigation, writing – original draft, writing – review and editing, project administration, funding acquisition. **Mirene Tchekep:** investigation, data curation, methodology, resources. **Jean Baptiste Boumsong:** methodology, investigation, data curation, project administration, resources, **s**upervision. **Jean Gustave Tsiagadigui:** resources, data curation, investigation, project administration, methodology, supervision. **Odette Dzemo Kibu:** writing – review and editing, supervision. **Fanny Nadia Dissak‐Delon:** investigation, methodology, project administration, data curation, supervision, resources. **Rasheedat Oke:** writing – review and editing, supervision, project administration. **Catherine Juillard:** writing – review and editing, project administration, supervision, conceptualization, investigation. **Alain Chichom‐Mefire:** conceptualization, investigation, supervision, project administration, writing – review and editing. **S. Ariane Christie:** conceptualization, investigation, writing – review and editing, project administration, supervision.

## Funding

Work supported by National Institutes of Health (5R21TW010453‐03, D43TW009343) and University of California Global Health Institute.

## Consent

Informed consent was obtained from all individual participants included in the study. The study was approved by the appropriate institutional review board. This manuscript is based on an abstract presentation at the ninth Owen H Wangensteen Scientific Forum of the American College of Surgeons Clinical Congress, October 2023, in Boston, MA.

## Conflicts of Interest

The authors declare no conflicts of interest.

## Data Availability

The data that support the findings of this study are available on request from the corresponding author. The data are not publicly available due to privacy or ethical restrictions.

## References

[1] S. M. Graham , L. Chokotho , N. Mkandawire , et al. “Injury: A Neglected Global Health Challenge in Low‐Income and Middle‐Income Countries,” Lancet Global Health 13, no. 4 (2025): e613–e615, 10.1016/s2214-109x(25)00074-9. 40155097

[2] Equi‐Trauma Collaborative . M. L. Odland , A. M. Abdul‐Latif , et al., “Equitable Access to Quality Trauma Systems in Low‐Income and Middle‐Income Countries: Assessing Gaps and Developing Priorities in Ghana, Rwanda and South Africa,” BMJ Global Health 7, no. 4 (2022): e008256, 10.1136/bmjgh-2021-008256. PMC9003614 35410954

[3] P. Frankiewicz , Y. Sawe , F. Sakita , et al. “Financial Toxicity and Acute Injury in the Kilimanjaro Region: An Application of the Three Delays Model,” PLoS One 19, no. 8 (2024): e0308539, 10.1371/journal.pone.0308539. 39213278 PMC11364231

[4] S. A. Christie , D. Dickson , S. N. Mbeboh , et al. “Association of Health Care Use and Economic Outcomes After Injury in Cameroon,” JAMA Network Open 3, no. 5 (2020): e205171, 10.1001/jamanetworkopen.2020.5171. 32427321 PMC7237963

[5] N. P. Raykar , R. R. Yorlets , C. Liu , et al. “The How Project: Understanding Contextual Challenges to Global Surgical Care Provision in Low‐Resource Settings,” BMJ Global Health 1, no. 4 (2016): e000075, 10.1136/bmjgh-2016-000075. PMC5321373 28588976

[6] A. Sarfraz , M. Khalil , S. Woldesenbet , et al., “Association of Discharge Against Medical Advice With Surgical Outcomes and Healthcare Cost,” Journal of the American College of Surgeons (2025). 10.1097/XCS.0000000000001469 40568963

[7] D. J. Zheng , M. T. Yost , L. N. Mbuh , et al. “From Targets to Solutions: Implementing a Trauma Quality Improvement Bundle in Cameroon,” Injury 55, no. 9 (2024): 111625, 10.1016/j.injury.2024.111625. 38772755 PMC12009632

[8] O. C. Nwanna‐Nzewunwa , S. A. Christie , M. Carvalho , et al., “Analysis of a National Trauma Registry in Cameroon: Implications for Prehospital Care Strengthening,” Panamerican Journal of Trauma, Critical Care and Emergency Surgery 7 (2018): 133–142, 10.5005/jp-journals-10030-1216.

[9] D. J. Zheng , L. N. Mbuh , R. Oke , et al., “Preventability of Injury‐Related Morbidity and Mortality at Four Hospitals in Cameroon: A Systematic Approach to Trauma Quality Improvement,” World Journal of Surgery (2024). 10.1002/wjs.12303 PMC11563858 39095973

[10] K. B. Japiong , G. Asiamah , E. Owusu‐Dabo , et al. “Availability of Resources for Emergency Care at a Second‐Level Hospital in Ghana: A Mixed Methods Assessment,” Afr J Emerg Med 6, no. 1 (2016): 30–37, 10.1016/j.afjem.2015.06.006. 30456061 PMC6233235

[11] Z. Bredow , Z. Corbett , M. M. Tarawally , et al. “Emergency Care Capacity in Sierra Leone: A Multicentre Analysis,” Afr J Emerg Med 14, no. 1 (2024): 58–64, 10.1016/j.afjem.2024.01.003. 38348097 PMC10859259

[12] T. Obembe and S. Fonn , “Affording Unavoidable Emergency Surgical Care – The Lived Experiences and Payment Coping Strategies of Households in Ibadan Metropolis, Southwestern Nigeria,” PLoS One 15, no. 5 (2020): e0232882, 10.1371/journal.pone.0232882. 32433652 PMC7239385

[13] J. J. Khan , F. Sehrin , Z. Quayyum , A. R. Sarker , and M. S. Rahman , “Factors Affecting Out‐of‐Pocket Expenditures for Chronic and Acute Illnesses in Bangladesh,” PLoS One 20, no. 4 (2025): e0320429, 10.1371/journal.pone.0320429. 40202947 PMC11981217

[14] E. Calvello , T. Reynolds , J. M. Hirshon , et al. “Emergency Care in Sub‐saharan Africa: Results of a Consensus Conference,” Afr J Emerg Med 3, no. 1 (2013): 42–48, 10.1016/j.afjem.2013.01.001.

[15] P. K. Machona , J. M. Zulu , M. Makasa , E. Meland , and T. Mildestvedt , “Are We Ready? Emergency Unit Capacity at Selected District Level Hospitals in Lusaka Province, Zambia: Barriers and Facilitators for Improving Trauma Care: A Mixed Methods Approach,” PLOS Global Public Health 5 (2025): e0004382, 10.1371/journal.pgph.0004382. 40344169 PMC12064030

[16] P. Nzasabimana , A. Ignatowicz , B. T. Alayande , et al. “Barriers to Equitable Access to Quality Trauma Care in Rwanda: A Qualitative Study,” BMJ Open 13, no. 9 (2023): e075117, 10.1136/bmjopen-2023-075117. PMC10546151 37770259

[17] T. E. Chao , K. Chu , T. C. Hardcastle , et al. “Trauma Care and Its Financing Around the World,” Journal of Trauma and Acute Care Surgery 97, no. 5 (2024): e60–e64, 10.1097/ta.0000000000004448. 39330943

[18] M. A. Franke , A. Neumann , K. Nordmann , et al. “Direct Patient Costs for Drugs and Consumables at Fifteen Health Facilities in Southern Madagascar, A Secondary Analysis of Patient Invoices,” PLoS One 19, no. 10 (2024): e0311253, 10.1371/journal.pone.0311253. 39388443 PMC11469595

[19] S. Hirner , J. Dhakal , M. C. Broccoli , M. Ross , E. J. Calvello Hynes , and C. B. Bills , “Defining Measures of Emergency Care Access in Low‐Income and Middle‐Income Countries: A Scoping Review,” BMJ Open 13, no. 4 (2023): e067884, 10.1136/bmjopen-2022-067884. PMC10111883 37068910

[20] H. K. H. Wesson , N. Boikhutso , A. M. Bachani , K. J. Hofman , and A. A. Hyder , “The Cost of Injury and Trauma Care in Low‐ and Middle‐Income Countries: A Review of Economic Evidence,” Health Policy and Planning 29, no. 6 (2014): 795–808, 10.1093/heapol/czt064. 24097794 PMC4153302

[21] Towards universal health coverage in the WHO African Region: tracking financial protection . Brazzaville: WHO African Region, 2024

[22] K. Werner , T. K. Lin , N. Risko , M. Osiro , J. Kalanzi , and L. Wallis , “The Costs of Delivering Emergency Care at Regional Referral Hospitals in Uganda: A Micro‐Costing Study,” BMC Health Services Research 21, no. 1 (2021): 232, 10.1186/s12913-021-06197-7. 33726738 PMC7961167

[23] J. L. Dieleman , T. Templin , N. Sadat , et al. “National Spending on Health by Source for 184 Countries Between 2013 and 2040,” Lancet 387, no. 10037 (2016): 2521–2535, 10.1016/s0140-6736(16)30167-2. 27086174

[24] P. A. Shah , S. A. Christie , G. Motwani , et al. “Financial Risk Protection and Hospital Admission for Trauma in Cameroon: An Analysis of the Cameroon National Trauma Registry,” World Journal of Surgery 44, no. 10 (2020): 3268–3276, 10.1007/s00268-020-05632-w. 32524159

[25] C. Asahngwa , O. D. Kibu , N. V. Ngo , et al. “Hospital Detention for the Inability to Pay: A Qualitative Study of Patient Experiences in Cameroon,” Journal of Surgical Research 290 (2023): 257–265, 10.1016/j.jss.2023.05.011. 37315440

[26] F. N. Dissak‐Delon , K. O’Connor , M. T. Yost , et al. “Do Deferred Emergency Payment Programmes Increase Use of Injury Care Services in Cameroon? A Trauma Registry Analysis,” BMJ Global Health 10, no. 3 (2025): e017760, 10.1136/bmjgh-2024-017760. PMC11938216 40132809

[27] C. I. Penda , C. E. Ekoube , R. M. Betoko , et al. “Deferred Recovery of Health Expenditures for Pediatric Life‐Threatening Emergencies in a Resource‐Limited Setting: Alternative Before Achieving Universal Health Coverage in Cameroon in Central Africa,” PLoS One 20, no. 6 (2025): e0322615, 10.1371/journal.pone.0322615. 40472071 PMC12140392

[28] L.‐Y. Wong , S. Hussain , M. Labib , et al. “Pilot Implementation Projects in Low‐ and Middle‐Income Countries to Guide Surgical Quality Improvement Using Best Practice Recommendations,” Front Heal Serv 5 (2025): 1423429, 10.3389/frhs.2025.1423429. PMC12209354 40599323

